# KYNURENINE PATHWAY INHIBITORS COMBINED WITH NAD+ PRECURSORS INCREASES LONGEVITY GENE EXPRESSION IN DROSOPHILA

**DOI:** 10.1093/geroni/igad104.3448

**Published:** 2023-12-21

**Authors:** Adam Said, Qiao Huang, Yuqiong Wu, Jeremy Walston, Reyhan Westbrook, Peter M Abadir

**Affiliations:** Emory College of Arts and Sciences, Atlanta, Georgia, United States; Johns Hopkins University School of Medicine, Baltimore, Maryland, United States; Johns Hopkins University School of Medicine, Baltimore, Maryland, United States; Johns Hopkins University, Baltimore, Maryland, United States; Johns Hopkins University School of Medicine, Baltimore, Maryland, United States; Johns Hopkins University School of Medicine, Baltimore, Maryland, United States

## Abstract

With rates of aging-related functional decline rising, the need for novel intervention strategies to extend lifespan has increased. In humans, the kynurenine pathway (KP) metabolizes tryptophan and produces nicotinamide adenine dinucleotide (NAD+). KP is correlated negatively with longevity and increased KP metabolite concentrations decrease the average and maximum lifespans in flies. However, genetic and pharmacological inhibition of tryptophan metabolism and dietary supplementation of NAD+ precursors increase the average and maximum lifespans in flies. To comprehensively explore the effects of KP manipulation, we fed DGRP_229 Drosophila males diets infused with ⍺-MT (KP inhibitor), nicotinamide (NAM, an NAD+ precursor), or the combination of NAM and ⍺-MT. We found that all treatments resulted in increased average and maximum lifespans, with the combination treatment displaying the greatest increase compared to the control of 30% (P< 0.0001). The combination treatment resulted in an average lifespan increase of 15% in relation to ⍺-MT alone (P< 0.0001) and 18% in comparison to NAM alone (P< 0.0001). To gain more insight into the biological mechanisms through which different treatments affect lifespan, we performed qRT-PCR on 11 longevity genes in Drosophila. We found that the combination treatment significantly increased the expression of gigas (Gig) (P=0.03), which interacts with Tsc1 to control cellular growth by regulating insulin and TOR signaling pathways, and sirtuin 1 (Sirt1) (P=0.03), an NAD+-dependent histone deacetylase. Our results demonstrate that the combined treatment of KP inhibition and NAD+ precursor elicits a transcription response similar to other lifespan-enhancing treatments and could be used as an alternative intervention method.

